# Color Stability of Three Ceramics After Thermocycling in Coffee, Black Tea, Cola, and Water: An In Vitro Study

**DOI:** 10.1155/ijod/6965595

**Published:** 2025-08-21

**Authors:** Dina Maleki, Donya Maleki, Arayeh Maleki, Helia Zare, AmirHossein SohrabiFar

**Affiliations:** ^1^Dental Sciences Research Center, School of Dentistry, Guilan University of Medical Sciences, Rasht, Iran; ^2^Dental Research Center, School of Dentistry, Tehran University of Medical Sciences, Tehran, Iran; ^3^Dental Sciences Research Center, School of Dentistry, Guilan University of Medical Sciences, Rasht, Iran

**Keywords:** ceramic, color, dental material

## Abstract

**Background:** Ceramic restorations have become a cornerstone of modern dentistry. Color stability is crucial for dental ceramics to maintain the esthetic appearance of restorations over time.

**Objectives:** Given the widespread consumption of staining agents like coffee, tea, and cola, this study aimed to assess the color stability of three ceramics after thermocycling in different solutions.

**Materials and Methods:** In this in vitro study, 120 samples of Vita Suprinity PC, IPS e.max, and InCoris ZI (40 samples each) were prepared as disks with 1 mm thickness and 10 mm diameter in A2 shade and glazed per manufacturer guidelines. The 10 samples of each group underwent 5000 thermocycles in coffee, black tea, cola, or tap water (5 and 55°C) with a dwell time of 30 s and a transfer time of 10 s simulating approximately 6 months of clinical use. To assess color change, *ΔE* was calculated using a calibrated spectrophotometer (X Rite, sp60 series, USA). *L* (for the lightness/darkness), *a* (for redness/greenness), and *b* (for yellowness/blueness) were measured before and after immersing. The data were analyzed using ANOVA with SPSS version 26.0. at the significance level of 0.05.

**Results:** According to the results in all three groups, *∆E* was significantly higher in the coffee subgroup compared to the black tea, cola, and water subgroups; was significantly higher in the black tea subgroup compared to the cola and water subgroups; and was significantly higher in the cola subgroup than the water subgroup (*p*  < 0.001). In the comparison of all four solutions, the color change in the Vita Suprinity PC samples was more significant compared to IPS e.max and InCoris ZI samples; and was more significant in IPS e.max than InCoris ZI (*p*  < 0.001). ∆*E* values for Vita Suprinity PC and IPS e.max were perceptible (*∆E* > 1) but clinically acceptable (*∆E* < 3.7), while InCoris ZI's *∆E* was imperceptible (*∆E* < 1).

**Conclusion:** The color stability of monolithic zirconia was more than lithium disilicate in coffee, black tea, cola, and water solutions. The color of lithium disilicate was more stable than zirconia-reinforced lithium silicate in all staining solutions. Coffee, black tea, and cola consumption can affect ceramic restorations' color change. Coffee has a more staining effect among different beverages.

## 1. Introduction

Ceramic restorations are a cornerstone of modern dentistry, valued for their biocompatibility, durability, and esthetic properties [1]. Dental ceramics, including feldspathic, leucite-reinforced, lithium disilicate, alumina-based, and zirconia, offer distinct characteristics tailored to specific clinical applications [2–4]. Feldspathic ceramics, composed primarily of silica (SiO_2_) and alumina, have a glassy structure that provides excellent translucency, making them ideal for veneers and anterior crowns due to their ability to mimic natural enamel [4, 5]. However, their low fracture resistance and brittleness limit their use in high-stress areas, such as posterior restorations or in patients with bruxism [5]. Leucite-reinforced ceramics incorporate leucite crystals into the glassy matrix, enhancing mechanical strength, while maintaining acceptable esthetics [4, 6]. These ceramics are commonly used for inlays, onlays, and anterior crowns, but their moderate strength makes them susceptible to chipping and unsuitable for high-load posterior restorations [6].

Lithium disilicate ceramics, such as IPS e.max, feature a glassy matrix reinforced with lithium disilicate crystals, offering a high flexural strength of 360–400 MPa and excellent translucency [4, 7]. These properties make them versatile for crowns, veneers, inlays, and onlays in both anterior and posterior regions [7]. However, their glassy matrix increases susceptibility to staining, and they require precise bonding techniques to prevent fracture under extreme occlusal forces [7]. Alumina-based ceramics, composed of aluminum oxide (Al_2_O_3_) crystals, have a highly crystalline structure with minimal glass content, providing superior mechanical strength for posterior crowns and fixed dental prosthesis frameworks [8]. Their limited translucency, however, restricts their use in highly esthetic zones, and their reduced adhesion capabilities often necessitate mechanical retention [4, 8]. Zirconia ceramics, such as InCoris ZI, are composed of zirconium dioxide (ZrO_2_) stabilized with yttrium oxide (Y_2_O_3_), offering exceptional fracture resistance (up to 1200 MPa) and biocompatibility [9–11]. Used for crowns, bridges, implant abutments, and frameworks, zirconia is often fabricated via CAD/CAM technology [11]. Monolithic zirconia enhances durability, while layered zirconia improves esthetics, though its lower translucency compared to glass-based ceramics and potential to wear opposing teeth remain challenges [11]. Zirconia-reinforced lithium silicate (e.g., Vita Suprinity PC) combines a glassy matrix with zirconia particles, balancing strength, and esthetics but with moderate stain resistance [4, 5, 11].

Dental ceramics are integral to restorative dentistry, with zirconia and lithium disilicate being among the most commonly used, accounting for approximately 50% and 25% of restorations, respectively [3, 6, 11, 12]. This study focuses on these frequently used ceramics to assess their performance in clinical scenarios. The growing demand for ceramics reflects an increasing emphasis on dental esthetics, as patients seek restorations that match the natural appearance of their teeth [13]. Color stability is critical, particularly for anterior restorations, where even minor discoloration can impact patient confidence and satisfaction [14].

Color stability is influenced by intrinsic factors, such as material composition and surface porosity, and extrinsic factors, including dietary stains, smoking, plaque accumulation, and exposure to hot or cold beverages [15–17]. The type of ceramic significantly affects its stain resistance; for instance, porcelain ceramics are more prone to color changes due to their porous glassy matrix [15]. Fabrication quality, ceramic thickness, cementation type, and surface texture also play roles [16]. Coffee, black tea, and cola, widely consumed globally, are major contributors to extrinsic staining due to their chromogenic compounds [18–23]. Coffee contains tannins and polyphenols that adhere to surfaces, causing discoloration [24–28]. Black tea, rich in tannins (11%–15% by weight), similarly promotes staining [25–28] Cola beverages, with caramel coloring (E150) and acidic components (pH ~ 2.5), can erode surfaces, increasing stain susceptibility [29–31]. These beverages are particularly relevant due to their high consumption rates, with coffee and tea consumed in millions of kilograms annually and cola served billions of times daily [19–23].

Previous studies, such as Gheytasi et al. [32], Sayed et al. [33], and Qaraghuli et al. [34], evaluated ceramic color stability after immersion in staining solutions but did not use thermocycling, limiting their clinical relevance. Thermocycling, which simulates oral temperature fluctuations (e.g., 5–55°C), better replicates clinical conditions by inducing thermal stresses that affect surface roughness, microstructure, and pigment penetration [35–37]. These stresses can reveal weaknesses in the ceramic glaze or surface, increasing stain susceptibility, which immersion tests may not detect [35–37]. Limited studies, such as Khomprang et al. [38] and Demirel et al. [39], have assessed color stability after thermocycling, but only in coffee, leaving gaps in understanding the effects of other common beverages.

To address these limitations, this study evaluated the color stability of Vita Suprinity PC, IPS e.max, and InCoris ZI after 5000 thermocycles in coffee, black tea, cola, and tap water. These ceramics were chosen for their frequent clinical use, and the beverages reflect common dietary habits. The null hypothesis was that no significant differences exist in color stability among the three ceramics in these solutions.

## 2. Methods

This in vitro study aimed to assess the color stability of three ceramic materials (Vita Suprinity PC, IPS e.max, and InCoris ZI) following thermocycling in coffee, black tea, cola, and water. A total of 120 ceramic samples were prepared; 40 samples of zirconia-reinforced lithium silicate (Vita Suprinity PC, Vita Zahnfabrik, Germany), 40 samples of lithium disilicate (IPS e.max CAD [IPC], Ivoclar Vivadent, Liechtenstein), and 40 samples of monolithic zirconia (InCoris ZI, Dentsply, Sirona Germany).

The ceramic blocks were sectioned using a diamond disk operating at 3000 rpm (Buehler Co MECATOM, T201A, Low-speed precision cutting machine, USA). Samples were prepared as disks with 1 mm thickness and 10 mm diameter to provide sufficient surface area for accurate spectrophotometric measurements while minimizing edge effects, per standard protocols. The samples were in A2 shade for its common use in clinical practice due to its natural appearance and for comparability with prior studies. All samples were dried and sintered according to manufacturer instructions as follows; the sintering process of Vita Suprinity PC LS began at a standby temperature of 400°C, with the temperature rising at 55°C/min until it reached 840°C. The samples underwent a cooling time of 8 min, with vacuum stages applied at 410 and 840°C. IEC required a standby temperature of 403°C, with a temperature rise rate of 90°C/min, reaching a sintering temperature of 820°C. Cooling lasted for 7 min and 10 s, with vacuum stages applied at 550 and 820°C. The sintering process of InCoris ZI started at 300°C, with a temperature rise rate of 8°C/min, reaching a higher sintering temperature of 1300°C. The cooling time was extended to 60 min, and no vacuum stages were applied.

After sintering, all samples were glazed according to manufacturer guidelines. Vita Suprinity PC LS samples were glazed using Vita Akzent Plus Glaze Paste at 800°C for 5 min with a heating rate of 80°C/min. IEC samples were glazed with a 1:1 mixture of IPS Empress Glaze Paste and Empress Universal Glaze at 960°C for 2 min with a heating rate of 60°C/min. For InCoris ZI samples, High SLU was applied at 820°C for 4 min, with a heating rate of 65°C/min.

To measure the initial color stability, the samples were placed over a black background to eliminate interference from underlying surfaces and ensure consistent, standardized color measurements, per ISO 28642:2011. A calibrated spectrophotometer (4 mm measuring tip, X Rite, sp60 series, USA) was used to determine *L*_0_*⁣*^*∗*^ is for the lightness/darkness of a color, *a*_0_*⁣*^*∗*^ is redness (positive) or greenness (negative), and *b*_0_*⁣*^*∗*^ is yellowness (positive) or blueness (negative) before immersion. One black background was used for all the measurements.

In total 40 samples of each ceramic group were randomly divided into four subgroups (*n* = 10). In the first and second subgroups, the samples underwent 5000 thermocycles (5 and 55°C) in coffee and black tea, respectively. To prepare coffee solution, 15 g coffee (NESCAFÉ classic, Nestlé Company, Switzerland) was dissolved in 200 mL boiling distilled water (Teb Shimi, Iran). The pH of the solution was measured using a pH meter (ISTEK, NeoMet 240-L, Seoul, South Korea). The pH of the coffee solution was 4.95. Coffee solution contained acids (chlorogenic acid, quinic acid, citric acid, and malic acid), caffeine, carbohydrates, lipids, proteins, amino acids, and phenolic compounds (ferulic acid and caffeic acid), according to the manufacturer. The black tea solution was prepared by dissolving 2 g of tea powder (Golestan, Iran) in 200 mL boiling distilled water (Teb Shimi, Iran). The pH was 5.01 measured by a pH meter (ISTEK, NeoMet 240-L, Seoul, South Korea). Black tea solution contained polyphenols (catechins, theaflavins, and thearubigins), caffeine, amino acids, l-theanine, tannins, and minerals, according to the manufacturer. In the third subgroup, the samples underwent 5000 thermocycles (5 and 55°C) in 200 mL cola (Coca-Cola, Coca-Cola Co, Istanbul, Turkey). The pH was 2.5 measured by a pH meter (ISTEK, NeoMet 240-L, Seoul, South Korea). Cola contained sweeteners, acidulants, carbonation (carbon dioxide and carbonic acid), flavorings, preservatives (sodium benzoate), caffeine, coloring agents, and water. In the fourth subgroup, the samples underwent 5000 thermocycles (5 and 55°C) in 200 mL water (tap water). The pH was 7.2 using a pH meter (ISTEK, NeoMet 240-L, Seoul, South Korea).

To perform 5000 thermocycles (5 and 55°C), a dwell time of 30 s and a transfer time of 10 s were considered. This thermocycling protocol simulates 6 months of clinical use in the oral environment. After thermocycling, the samples were brushed 10 times with toothpaste under running water. The color stability parameters (*L*_1_*⁣*^*∗*^, *a*_1_*⁣*^*∗*^, and *b*_1_*⁣*^*∗*^) were measured again after immersion. *ΔE* was calculated based on the following equation:

  $$\begin{array}{c} \Delta E^{*}=\frac{{\left(\Delta L^{*}\right)}^{2}+{\left(\Delta a^{*}\right)}^{2}+{\left(\Delta b^{*}\right)}^{2}}{2}. \end{array}$$

The CIE-Lab-based *∆E* formula was used to ensure comparability with prior studies (e.g., Gheytasi et al. [32]; Sayed et al. [33], though *∆E*00 is more sensitive to human perception. The data were analyzed using repeated measure ANOVA and Tuckey's post hoc test with SPSS version 26.0 (IBM Corp, Armonk, NY, USA). The analysis considered coloring agents (coffee, black tea, cola, and water) and ceramic type as between-subject factors. A confidence interval of 95% was used, and *p*  < 0.05 was considered statistically significant.

## 3. Results

**Figure 1:**
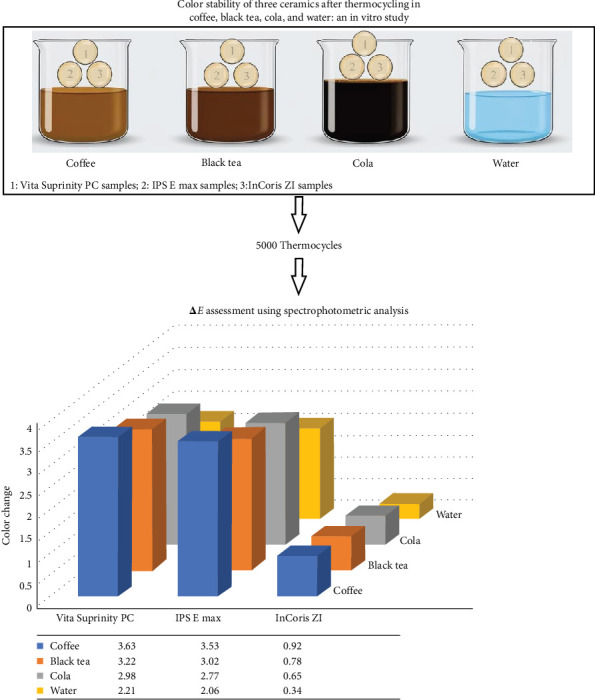
The color stability of monolithic zirconia was more than lithium desilicate in coffee, black tea, cola, and water solutions. The color of lithium desilicate was more stable than zirconia-reinforced lithium silicate in all staining solutions. Coffee, black tea, and cola consumptions can affect ceramic restorations' color change. Coffee has a more staining effect among different beverages.

**Figure 2:**
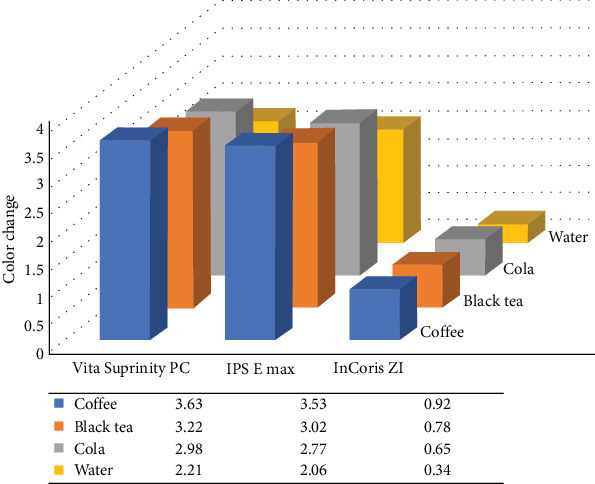
Color change (*∆E*) of Vita Suprinity PC, IPS E max, and InCoris ZI ceramics in coffee, black tea, cola, and water solutions measured against a black background.

In this study, 120 samples made of three different ceramics (Vita Suprinity PC, IPS E max, and InCoris ZI) were subjected to four solutions (coffee, black tea, cola, and water solutions). (Figure 1) The measurements of *∆a, ∆b, and ∆L* are presented in Table 1. The data analysis revealed that all four solutions caused significant color change (*∆E*) in three studied ceramics (Figure 2). According to the results in the three studied ceramics, *∆E* was significantly higher in the coffee solution compared to black tea, cola, and water solutions. In the three studied ceramics, *∆E* was significantly higher in the black tea solution compared to the cola and water solutions. In the three studied ceramics, *∆E* was significantly higher in the cola solution than the water solution.  Table 2 presents the analysis results of two-by two comparisons of the color change of the three studied ceramics according to coffee, black tea, cola, and water solutions.

The color changes caused by coffee, black tea, cola, and water solutions were significantly higher in the Vita Suprinity PC samples compared to IPS e.max and InCoris ZI samples and were significantly higher in the IPS e.max. samples compared to InCoris ZI.  Table 3 presents the analysis results of two-by two comparisons of the color change of coffee, black tea, cola, and water solution, according to Vita Suprinity PC, IPS E max, and InCoris ZI ceramics.

## 4. Discussion

In the current study, the most commonly used beverages in a Middle Eastern population were selected; coffee, black tea, and cola. Coffee is one of the most widely consumed beverages globally, with significant consumption patterns observed both worldwide and within the Middle East [18, 19]. According to the International Coffee Organization, global coffee consumption has been steadily increasing, with millions of 60 kg bags consumed annually [18, 19]. In the Middle East, coffee holds substantial cultural significance, contributing to its widespread consumption [39]. The region has witnessed a notable increase in coffee consumption in recent years, driven by factors, such as changing lifestyles and a growing preference for specialty coffee [40]. This rising trend is further evidenced by the expansion of the ready-to-drink coffee market in the Middle East, which is expected to grow at a compound annual growth rate of 10.48% from 2024 to 2030 [41]. The increasing number of branded coffee shops in the Middle East also reflects the region's growing coffee culture. The total market grew by 10.5% over the last 12 months, reaching 8874 outlets, becoming a key geography for major international coffee chains and new domestic operators [42]. These statistics highlight the significant role coffee plays both globally and within Middle Eastern societies, with consumption patterns influenced by cultural traditions and evolving consumer preferences [18, 19, 39–42].

Black tea is also one of the most consumed beverages worldwide, with significant consumption in both global and Middle Eastern populations [20–22]. Globally, tea consumption reached approximately 7.3 billion kilograms in 2023, with black tea being a substantial portion of this consumption [43]. In the Middle East, black tea holds a prominent place in daily life and culture [44]. Countries like Turkey and Iran have some of the highest per capita tea consumption rates globally, with Turkey leading at 3.16 kg per person and Iran at 1.50 kg per person annually [44]. The strong tea-drinking culture in the Middle East is further evidenced by the significant market presence of black tea in the region [45]. The Middle East black tea market is expected to grow at a compound annual growth rate of nearly 11% from 2024 to 2029, driven by cultural traditions and a strong preference for black tea varieties [44, 45]. This deep-rooted cultural affinity for black tea underscores its importance in both global and Middle Eastern contexts [20–22, 43–45].

Cola beverages was also included in the current study. Cola, particularly those produced by major companies, are among the most consumed soft drinks globally [23, 24]. Cola products are served approximately 1.9 billion times daily worldwide, highlighting their extensive reach and popularity [46]. In the Middle East, the consumption of carbonated soft drinks, including cola, is substantial [46]. In 2021, the combined consumption of packaged beverages in the Middle East was nearly 118 billion liters, with a projected annual growth rate of 2.4% expected to reach 127.1 billion liters by 2024 [46]. This significant consumption underscores the cultural integration and preference for cola beverages in both global and Middle Eastern markets [23, 24, 46].

The current study only considered a limited range of staining agents, focusing on beverages commonly consumed by a broad population in Middle East. The first limitation of this study is the fact that red wine as a commonly used beverages was not included in the current study as this study was performed in an Islamic country in which the consumption of red wine is illegal and the authors were unable to prepare red wine for the purpose of this study. Future studies should expand the scope of testing to include other common dietary substances with staining potential, such as red wine, berries, and spices.

The results of this study showed that the color change of all three materials (Vita Suprinity PC, IPS e.max, and InCoris ZI) was significantly higher in coffee solution followed by black-tea, cola, and water, respectively.

As for coffee and black tea solutions, they have staining properties due to their chemical composition specially tannins [47]. Tannins are yellow-brown polyphenol molecules with low polarity and have strong chromogenic properties, meaning they can bind to surfaces, like dental materials and cause discoloration over time [48–50]. The interaction between tannins and saliva can further facilitates the color change of ceramics [51].

The concentration of tannins varies significantly between black tea and coffee, with black tea generally containing a higher amount [52–54]. Black tea typically contains around 11%–15% tannins by dry weight [52–54]. The high concentration of tannins in black tea comes from the oxidation process that occurs during its fermentation, making it one of the most tannin-rich beverages [52–54]. Coffee has approximately 4%–5% tannins by dry weight. The tannin content in coffee mainly comes from chlorogenic acids, which are a type of polyphenol but less potent in terms of the staining power compared to the tannins in black tea [52–54].

It should be noted that, the discoloration of ceramics from coffee, despite its lower tannin content compared to black tea, is largely due to other compounds and the nature of the beverage itself [55–57]. Coffee contains a significant number of dark pigments known as melanoidins, which are created during the roasting process [55–57]. These melanoidins are highly adhesive to porous surfaces like ceramic, causing discoloration. Also, coffee is more acidic than most teas. Its acidic nature can slightly wear down the surface of ceramics, making them more susceptible to absorbing stains from pigments over time [55–57].

As for Cola solution, the acidic nature of Cola, with a pH around 2.5, leads to erosion and surface roughness on ceramic materials enhancing the ceramic's ability to absorb pigments from the beverage [58–60]. Cola also contains coloring agents, such as caramel which in turn contributes to staining. Additionally, the sugar content in Cola may promote the retention of pigments on ceramic surfaces, although the key factor remains the acidic nature and the presence of colorants that penetrate microporosities formed on the ceramic surface [58–60].

As for water solution, water promotes the breakdown of resin matrices of dental ceramic, leading to increased staining from pigments present in beverages or food [61–63]. The interaction between water and ceramic surfaces can lead to roughness, making the material more susceptible to staining. Additionally, microcracks that form from prolonged exposure to moisture can worsen discoloration by trapping stains more easily [61–63].

Therefore, it can be concluded that, coffee and black tea cause discoloration for containing tannins, cola can cause color change for its acidic nature and containing coloring agents, and water can cause discoloration by causing microcracks in the ceramics.

In the current study, the most commonly used ceramics in dentistry were selected. Zirconia ceramics, with unmatched strength and durability, dominate usage (50%) globally, despite challenges like limited translucency and wear on opposing teeth. Lithium disilicate ceramics balance strength and translucency, suitable for most restorations, though they require precise bonding, follow with 25%, showcasing their versatility in modern restorative dentistry [3, 6, 11, 64]. The results of this study also claimed that the color change (*∆E*) in the Vita Suprinity PC was more significant than IPS e.max and InCoris ZI, and more significant in IPS e.max than InCoris ZI.

The structure and composition of lithium disilicate and zirconia ceramics are integral to their color stability and clinical performance, as these properties directly influence how the materials interact with staining agents and environmental conditions [1–9]. Lithium disilicate ceramics are characterized by a dual-phase structure, consisting of elongated lithium disilicate (Li_2_Si_2_O_5_) crystals embedded within a glassy matrix. This glass–ceramic combination is engineered to optimize both mechanical and esthetic properties [3, 6, 7, 9]. The lithium disilicate crystals, which can make up approximately 60%–70% of the material by volume, are responsible for its high strength (360–400 MPa) and fracture resistance. These crystals act as reinforcement, distributing stress across the material, and minimizing the risk of fracture under occlusal forces [3, 6, 7, 9].

The glassy matrix, on the other hand, is composed primarily of silica (SiO_2_) and other glass modifiers, such as potassium oxide (K_2_O) and Al_2_O_3_, which enhance translucency by mimicking the light transmission properties of natural enamel [3, 9] However, this glassy phase introduces certain vulnerabilities [3]. Its inherent porosity and hydrophilic nature facilitate fluid absorption, making the material more susceptible to staining [4]. Over time, the surface of lithium disilicate ceramics can develop microporosities and minor imperfections due to wear, particularly under acidic conditions [4]. These imperfections act as pathways for pigments to penetrate, leading to discoloration. Furthermore, the protective glaze layer applied to the surface during manufacturing can wear down with brushing and thermocycling, exposing the underlying matrix to external agents [3, 4]. This degradation is particularly noticeable in environments with fluctuating temperatures or acidic pH levels, where the glassy phase can undergo chemical changes that enhance its affinity for chromogenic compounds [5].

In contrast, zirconia ceramics are predominantly polycrystalline, consisting of tightly packed ZrO_2_ crystals stabilized with Y_2_O_3_. The stabilization process creates a tetragonal phase at room temperature, which imparts exceptional mechanical properties to zirconia, including a flexural strength exceeding 1200 MPa and a high resistance to crack propagation [1–5]. Unlike lithium disilicate, zirconia lacks a glassy phase, which is a key factor in its superior resistance to staining and fluid absorption. The absence of a glassy matrix results in a dense, nonporous surface that resists pigment penetration even when subjected to acidic environments or prolonged thermocycling [6, 7].

At the molecular level, the highly organized crystalline structure of zirconia creates minimal surface irregularities, reducing the potential for pigment adherence [8, 9]. Additionally, zirconia exhibits a phenomenon known as transformation toughening, where stress at the material's surface induces a phase transformation from the tetragonal to the monoclinic phase [5, 7, 9]. This process not only prevents crack propagation but also contributes to the material's durability in challenging oral environments. However, zirconia's dense crystalline structure and lack of translucency can limit its esthetic application in anterior restorations, where high translucency is often desired [5, 7].

ZLS ceramics, such as Vita Suprinity PC, bridge the gap between these two materials by incorporating zirconia particles (approximately 10% by weight) into a lithium silicate glassy matrix. This hybrid composition enhances mechanical strength and reduces the porosity associated with traditional lithium disilicate ceramics [4, 5, 11]. However, the glassy phase in ZLS still makes it more susceptible to staining compared to monolithic zirconia. The zirconia particles provide some resistance to fluid absorption, but the material's overall color stability remains inferior to that of zirconia due to the presence of the glassy matrix [1, 3, 5, 6].

In summary, the molecular and structural differences between lithium disilicate and zirconia ceramics account for their distinct performance in terms of color stability and clinical applications [7, 11]. Lithium disilicate, with its glass-ceramic composition, offers superior translucency and esthetics but is more prone to staining due to its porous glassy matrix [6, 9, 11]. Zirconia, with its dense polycrystalline structure, resists staining and fluid absorption but sacrifices some translucency, making it less suitable for anterior restorations where esthetics are critical. ZLS ceramics attempt to combine the strengths of both materials but still inherit some of the staining vulnerabilities of their glassy phase. Understanding these compositional nuances is essential for selecting the appropriate material for specific clinical situations, balancing esthetic requirements with long-term durability [11].

Gheytasi et al. [32] evaluated the color stability of glazed monolithic zirconia after immersing in orange juice, black tea, and distilled water for 135 min/day for 24 days. The blocks were disk-shaped (10 mm × 2 mm) and in the shade of A2. *∆E* was measured to determine color stability. Their results showed that the black tea caused maximum color change compared to orange juice. Regarding the black tea, the study of Gheytasi et al. [32] reported similar findings as the current study. Black tea led to the highest color change in the current study following coffee solution. Gheytasi et al. [32] also reported that distilled water caused no significant color change. This finding is not in agreement with the current study. This inconsistency may be due to different methodology (immersing vs thermocycling) and used solutions. In the study of Gheytasi et al. [32], samples were immersed in distilled water, while in the current study, tap water was used for thermocycling. Regarding immersing and thermocycling, it should be stated that thermocycling simulates clinical conditions while immersing does not mimic clinical environment [36]. Regarding distilled water and tap water, as explained previously, water can lead to surface roughness of the dental ceramics increasing the chances of pigmentations. Tap water contains more minerals and particles compared to the distilled water which explains why the samples showed more color change against tap water in the current study compared to the distilled water in the study of Gheytasi et al. [32].

Sayed et al. [33] studied the color stability of IPS e.max and Vita Vita Suprinity PC and Vita Enamic disks (10 mm in diameter and 1.5 mm thick) immersed in coffee solution for 12 days. The *ΔE* measurement was used to report the color stability. Contrary to the current study, their findings showed that there is no significant difference between the color stability of included materials. Different disk thickness between the two studies (1.5 mm vs 1 mm) could be the reason for different results. The thickness of dental ceramics significantly affects their color stability and final appearance [65, 66]. Variations in thickness influence how light interacts with the material, impacting translucency, opacity, and the effect of underlying structures, such as the tooth or cement [65, 66]. Thicker ceramics increase light scattering, making them more opaque and less influenced by the underlying color [65, 66] This provides more consistent color stability and better masking of discolorations [65, 66]. On the other hand, thinner ceramics allow more light to pass through, enhancing translucency but making them more dependent on the color of the underlying tooth or cement [65, 66]. Subtle changes in the substrate or luting agent can significantly alter the perceived color of thin ceramics, compromising color stability [65, 66]. Also, it should be noted that Sayed et al. [33] immersed the disks into the coffee solution, meanwhile, the current study used the coffee thermocycling method; which can explain the different results. Thermocycling simulates clinical use in the oral environment while immersing does not mimic clinical environment [33].

Qaraghuli et al. [34] assessed the color stability of feldspathic ceramic (VITA BLICs), hybrid ceramic (VITA ENAMIC), zirconia-reinforced lithium silicate glass ceramic (VITA SUPRINITY PC), and composite resin (VITA-VMLC) after immersing in coffee, black tea and red wine for 72 h. The blocks were rectangular-shaped (10 mm × 12 mm × 2.5 mm) and in the shade of A2. The color change was assessed in their study by calculating the CIE-Lab system. They stated that the four studied restorative material showed color change significantly. The color change was more significant in composite resin group followed by hybrid ceramic, and zirconia-reinforced lithium silicate. Feldspathic ceramics had the lowest color change. According to the results, there was no significant difference among the three solutions. The results of Qaraghuli et al. [34] were unlike the current findings that the color change of all three materials (Vita Suprinity PC, IPS e.max, and InCoris ZI) was significantly higher in coffee solution followed by black-tea, cola, and water, respectively. Coffee and black tea material and preparation protocol were different between the two study. In the current study, NESCAFÉ classic and Golestan tea were used to prepare the coffee and black tea solutions, respectively. In the study of Qaraghuli et al. [34] Illy classic coffee (Illycaffe S.p.A., Trieste, Italy) and black tea with cardamom flavoring (Ahmad Tea London Ltd., Winchester Road, United Kingdom) were used to prepare the coffee and black solutions, respectively. Also, in the current study, color stability was assessed after 5000 thermocycles representing 6 months of clinical exposure [36]. While in the study of Qaraghuli et al. [34], color stability was measures after 72 h of immersion in the studied solutions. As stated previously, the immersion cannot stimulate oral condition like thermocycling.

Khomprang et al. [38] studied the color stability of 60 disks (14 mm × 16 mm × 1 mm) of IEC and IPS E.max ZirCAD Prime after 30,000 cycles of coffee thermocycling using *ΔE* measurements. Their study showed that there was no significant difference in *ΔE* between specimens. This finding was not in line with the current study. The cause of this inconsistency can be due to different thermocycle protocols. In the study of Khomprang et al. [38], samples underwent 30,000 cycles of coffee thermocycling, while in the current study, 5000 cycles of thermocycling were applied. Also, the use of ultrasonic cleaning, due to its ability to produce microcracks in the specimens, can be another origin for inconsistency between the two studies. Therefore, in the current study, the samples were cleaned using a tooth brush to prevent the formation of microcracks in the samples.

Demirel et al. [39] in 2022 assessed the color stability of three lithium silicate-based materials (CEREC Tessera, ALDS; IEC, LDS; Vita Vita Suprinity PC, ZLS) after 5000 cycles of coffee thermocycling. the specimens were disk-shaped (ø: 12 mm, thickness: 1.2 mm) and in A2 shade. The color change was reported using *ΔE00*. Their study showed that *ΔE00* values of tested materials were similar. The results of Demirel et al. [39] were not in line with the current study stating that the color change of Vita Suprinity PC was more significant than IPS e.max after thermocycling in coffee, black tea, cola, and water. This discrepancy may be due to different methodologies as in the study of Demirel et al. [39] the disks were 1.2 mm thick while in the current study, the disks were 1 mm thick. Also, in the study of Demirel et al. [39], color stability was measured using *ΔE00*, while in the current study CIE-Lab system (*ΔE*) was used.

*ΔE00* is more sensitive to human perception of color changes because it was designed as an improvement over the Δ*E* to better align with the way humans perceive color differences. The *ΔE00* formula takes into account factors, such as lightness, chroma, and hue interactions, as well as nonlinearities in human color perception. This allows *ΔE00* to provide more accurate assessments of perceptible color differences. In comparison, Δ*E* is a simpler formula that may not capture all the nuances of human perception, particularly in low chroma regions. Therefore, studies using *ΔE0*0 can more precisely assess whether color changes are noticeable to the human eye, leading to potentially different conclusions about the acceptability of color stability in dental materials. Using the CIE-Lab system in the current study was the second limitation of the current study [67–69].

Another limitation is the lack of scanning electron microscopy (SEM) analysis to evaluate surface changes after thermocycling in staining agents, which could elucidate surface roughness and microstructural alterations contributing to staining; this was not performed due to resource constraints. Future studies should incorporate SEM analysis to understand surface-related staining mechanisms.

The clinical thresholds for color stability in dentistry, measured by the *ΔE* formula, are well-documented in the literature guiding the clinicians and researchers in evaluating the perceptibility and acceptability of color changes in dental materials and restorations. ISO technical report (28642:2011:Dentistry Guidance on Color Measurement) provides comprehensive guidelines on color measurement in dentistry. It defines that the Δ*E* less than 1 is generally imperceptible to the human eye; the *ΔE* of 1–3.7 is clinically acceptable for most patients; and the Δ*E* more than 3.7 is noticeable for patients and is considered unacceptable in esthetically demanding cases, especially in the anterior region [70–72].

In the current study, the *ΔE* of Vita Suprinity PC and IPS e.max ceramics was above one, while the *ΔE* of InCoris ZI ceramic was below one in all four studied solutions. This finding indicates that the color changes caused by coffee, black tea, cola and water in the Vita Suprinity PC and IPS e.max ceramics were perceptible for human eye after 5000 cycles while this color change was imperceptible for the human eye in the InCoris ZI ceramic after 5000 cycles. The current study showed that the *ΔE* in all study groups in all solution was below 3.7 meaning that the color change of the three studied ceramics was clinically acceptable in the coffee, black tea, cola, and water solutions after 5000 of thermocycling. According to the results, it can be claimed that although all studied ceramics showed significant color change after 5000 thermocycles in the studies solutions, their color change was clinically acceptable.

This finding highlights the third limitation of the current study. This study assessed the color change of ceramics after 5000 thermocycles which represents 6 months of clinical use. More thermocycles (10,000, 20,000, 50,000, or 100,000 thermocycles) that simulate one, two, five, or 10 years of clinical exposure in oral mouth could result in more color change. As previous studies have assessed the color change of dental ceramics in oral cavity after a period of one to 10 years [72, 73].

The present study, like many in vitro investigations, has certain limitations that should be acknowledged. First, this study was conducted in a controlled laboratory environment using thermocycling to simulate the oral cavity. However, oral environment has additional factors, such as saliva, biofilm, variations in oral pH, and daily wear and tear, which were not fully replicated. Specially the absence of saliva and biofilm are significant limitations. Further studies in clinical environments are required. Also, future studies can test the influence of oral hygiene products like toothpaste and mouthwash on the color stability of dental ceramics and other dental materials.

## 5. Conclusion

In conclusion, this study provides valuable insights into the color stability of three widely used ceramic materials (Vita Suprinity PC, IPS e.max, and InCoris ZI) when exposed to common beverages. Monolithic zirconia (InCoris ZI) exhibited the least color change after 5000 thermocycles in coffee (NESCAFÉ Classic), black tea (Golestan), cola (Coca-Cola), and tap water, demonstrating superior color stability compared to lithium disilicate (IPS e.max) and zirconia-reinforced lithium silicate (Vita Suprinity PC). Lithium disilicate (IPS e.max) showed greater color stability than zirconia-reinforced lithium silicate (Vita Suprinity PC) in all four staining solutions. For clinical practice, InCoris ZI is an excellent choice for patients prioritizing long-term esthetics due to its superior stain resistance. IPS e.max offers reliable color stability, while Vita Suprinity PC, despite its esthetic appeal, showed greater staining, particularly in coffee, suggesting the need for surface optimization. All ceramics exhibited clinically acceptable color changes (*∆E* < 3.7), with InCoris ZI's changes being imperceptible (*∆E* < 1). Coffee caused the greatest staining, followed by black tea, cola, and tap water. These findings highlight that coffee, black tea, and cola consumption can affect ceramic restorations, with coffee having the most significant staining effect. Future studies should use *∆E*00 and SEM analysis to further explore color stability and surface changes.

## Figures and Tables

**Table 1:** *∆L*, *∆a*, and *∆b* of Vita Suprinity PC, IPS E max, and InCoris ZI ceramics in coffee, black tea, cola, and water solutions against a black background.

Parameters	Vita Suprinity PC				IPS E max				InCoris ZI			
	Coffee	Black tea	Cola	Water	Coffee	Black tea	Cola	Water	Coffee	Black tea	Cola	Water
*∆*L^a^	1.49 ± 1.78	1.21 ± 1.24	1.12 ± 1.81	1.07 ± 1.32	0.71 ± 1.24	0.66 ± 0.12	0.63 ± 0.86	0.51 ± 1.3	0.83 ± 0.37	0.78 ± 0.28	0.71 ± 0.34	0.56 ± 0.4
*∆a* ^b^	0.13 ± 0.66	0.09 ± 0.21	0.07 ± 0.11	0.08 ± 0.07	0.23 ± 0.87	0.18 ± 0.18	0.17 ± 0.62	0.15 ± 0.23	0.37 ± 0.03	0.20 ± 0.04	0.27 ± 0.07	0.21 ± 0.11
*∆*b^c^	1.84 ± 4.90	1.68 ± 4.72	1.53 ± 3.87	1.21 ± 2.73	2.41 ± 0.58	2.73 ± 0.82	2.68 ± 0.34	2.16 ± 5.4	0.89 ± 0.51	0.74 ± 0.03	0.67 ± 0.53	0.59 ± 0.57

^a^ *∆L* is for the lightness/darkness of a color.

^b^ *∆a* is for redness/greenness.

^c^∆*b* is for yellowness/blueness.

**Table 2:** The analysis results of two-by two comparisons of the color change of Vita Suprinity PC, IPS E max, and InCoris ZI ceramics according to coffee, black tea, cola, and water solutions.

Ceramics	Solutions	Coffee	Black tea	Cola	Water
Vita Suprinity PC	Coffee	—	0.028*⁣*^*∗*^	0.015*⁣*^*∗*^	0.001*⁣*^*∗*^
	Black tea	0.028*⁣*^*∗*^	—	0.047*⁣*^*∗*^	0.032*⁣*^*∗*^
	Cola	0.015*⁣*^*∗*^	0.047*⁣*^*∗*^	—	0.042*⁣*^*∗*^
	Water	0.001*⁣*^*∗*^	0.032*⁣*^*∗*^	0.042*⁣*^*∗*^	—

IPS E max	Coffee	—	0.041*⁣*^*∗*^	0.033*⁣*^*∗*^	0.025*⁣*^*∗*^
	Black tea	0.041*⁣*^*∗*^	—	0.048*⁣*^*∗*^	0.030*⁣*^*∗*^
	Cola	0.033*⁣*^*∗*^	0.048*⁣*^*∗*^	—	0.042*⁣*^*∗*^
	Water	0.025*⁣*^*∗*^	0.030*⁣*^*∗*^	0.042*⁣*^*∗*^	—

InCoris ZI	Coffee	—	0.049*⁣*^*∗*^	0.038*⁣*^*∗*^	0 < 0.001*⁣*^*∗*^
	Black tea	0.049*⁣*^*∗*^	—	0.042*⁣*^*∗*^	0 < 0.001*⁣*^*∗*^
	Cola	0.038*⁣*^*∗*^	0.042*⁣*^*∗*^	—	0 < 0.001*⁣*^*∗*^
	Water	0 < 0.001*⁣*^*∗*^	0 < 0.001*⁣*^*∗*^	0 < 0.001*⁣*^*∗*^	—

*⁣* ^*∗*^Significant finding according to ANOVA and Tukey's post hoc test.

**Table 3:** The analysis results of two-by two comparisons of the color change of coffee, black tea, cola, and water solutions according to Vita Suprinity PC, IPS E max, and InCoris ZI ceramics.

Solutions	Ceramics	Vita Suprinity PC	IPS E max	InCoris ZI
Coffee	Vita Suprinity PC	—	0.044*⁣*^*∗*^	0.001*⁣*^*∗*^
	IPS E max	0.044*⁣*^*∗*^	—	0.005*⁣*^*∗*^
	InCoris ZI	0.001*⁣*^*∗*^	0.005*⁣*^*∗*^	—

Black tea	Vita Suprinity PC	—	0.046*⁣*^*∗*^	0.001*⁣*^*∗*^
	IPS E max	0.046*⁣*^*∗*^	—	0.006*⁣*^*∗*^
	InCoris ZI	0.001*⁣*^*∗*^	0.006*⁣*^*∗*^	—

Cola	Vita Suprinity PC	—	0.049*⁣*^*∗*^	<0.001*⁣*^*∗*^
	IPS E max	0.048*⁣*^*∗*^	—	0.003*⁣*^*∗*^
	InCoris ZI	<0.001*⁣*^*∗*^	0.003*⁣*^*∗*^	—

Water	Vita Suprinity PC	—	0.041*⁣*^*∗*^	0.002*⁣*^*∗*^
	IPS E max	0.041*⁣*^*∗*^	—	0.005*⁣*^*∗*^
	InCoris ZI	0.002*⁣*^*∗*^	0.005*⁣*^*∗*^	—

*⁣* ^*∗*^Significant finding according to ANOVA and Tukey's post hoc test.

## Data Availability

The datasets supporting the findings of the current study are available from the corresponding author on reasonable request.
